# Predictors of Incident Heart Failure in Patients With Chronic Chagas Disease Cardiomyopathy

**DOI:** 10.1111/echo.70163

**Published:** 2025-04-28

**Authors:** Danton Machado da Cunha, Mauro Felippe Felix Mediano, Lorena dos Santos Marreto Rimolo, Andréa Rodrigues da Costa, Danilo Bento Diogo, Luiz Henrique Conde Sangenis, Henrique Horta Veloso, Marcelo Teixeira de Holanda, Alejandro Marcel Hasslocher‐Moreno, Ademir Batista da Cunha, Roberto Magalhães Saraiva

**Affiliations:** ^1^ Antonio Pedro University Hospital Fluminense Federal University Niterói RJ Brazil; ^2^ Clinical Research Laboratory in Chagas Disease Evandro Chagas National Institute of Infectious Diseases Oswaldo Cruz Foundation Rio de Janeiro RJ Brazil

**Keywords:** chagas disease, heart failure, progression, prognosis, strain

## Abstract

**Purpose:**

Patients with chronic Chagas cardiomyopathy (CCC) have a high mortality due to heart failure (HF). The aim of this study was to investigate clinical and echocardiographic predictors of incident HF in patients with CCC.

**Methods:**

Single‐center retrospective longitudinal observational study which included 176 adult patients (59.1% women; 53.9 ± 10 years old; mean left ventricular [LV] ejection fraction 62% ± 10%) at an early stage of CCC (electrocardiogram and/or wall motion changes but no HF). The primary outcome was incident HF. The association between studied parameters with incident HF was performed by competing‐risk survival regression models using the Fine and Gray method.

**Results:**

After a mean follow‐up of 8.8 ± 3.6 years, 42 patients progressed to HF (27.04 cases/1000 patient‐years). A model 0 adjusted for clinical and 2D‐Doppler echocardiographic parameters and for all‐cause mortality revealed diabetes mellitus (HR 4.91, 95% CI 1.67–14.4, *p* = 0.004), LV ejection fraction (HR 0.96, 95% CI 0.93–0.99, *p* = 0.022), and E’ velocity (HR 0.79, 95% CI 0.67–0.95, *p* = 0.01) as independently associated with incident HF. The addition of strain‐derived parameters to model 0 revealed that LV global circumferential strain (HR 0.83, 95% CI 0.78–0.89, *p* < 0.001) and left atrial booster contraction strain (HR 1.14, 95% CI 1.02–1.28, *p* = 0.022) were associated with incident HF.

**Conclusion:**

While most clinical parameters were not associated with incident HF in patients with CCC, echocardiographic parameters, including LV systolic and diastolic function and strain‐derived parameters, were associated with incident HF in patients with CCC. This knowledge can be very useful for planning the care and follow‐up of these patients.

## Graphical abstract

While most clinical parameters were not associated with incident heart failure (HF) in patients with chronic Chagas disease cardiomyopathy, echocardiographic parameters, including LV systolic and strain‐derived parameters, were associated with incident HF. This knowledge can be very useful for planning the care and follow‐up of these patients.

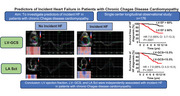

**Abbreviations**
CCC: chronic Chagas cardiomyopathy
CD: Chagas disease

## 1 Introduction

About 6–7 million people live with chronic Chagas disease (CD) throughout the world, mainly in Latin America [1]. However, CD has been diagnosed in non‐endemic countries, such as US, where at least 300 000 people live with chronic CD [2], and European countries, where the prevalence of CD among Latin American migrants is estimated to be 4.2% [3]. Among patients with chronic CD, 30% to 40% present chronic Chagas cardiomyopathy (CCC), which is diagnosed by typical changes in the electrocardiogram (ECG). Patients with CCC can complicate with left ventricular (LV) segmental or global systolic dysfunction, arrhythmias, cardioembolic stroke, sudden death, and heart failure (HF) with reduced ejection fraction [4, 5]. HF is present in about 10% of those with CCC [6], with CD being a major cause of HF in endemic countries ranging from 4% to 50%, depending on the study/registry [6, 7, 8]. Moreover, HF due to CD presents higher mortality [8, 9, 10] and stroke incidence [8] than HF related to other etiologies.

Although mortality predictors in chronic CD have been described [4, 5, 11, 12], to the best of our knowledge, no predictor of incident HF in CCC has been described. Risk factors for incident HF in the general population include coronary artery disease, diabetes mellitus, age, hypertension, smoking, male sex, body mass index, atrial fibrillation, LV hypertrophy, and valvular heart disease [13]. However, CD pathophysiology is characterized by a low‐grade chronic fibrosing myocarditis, related to the persistence of the *Trypanosoma cruzi* and immunological reactions within the myocardium, with consequent replacement of myocardium for fibrosis and compensatory hypertrophy of remnant myocytes [14, 15]. This process is probably independent from most of the described risk factors for incident HF, which justifies the search for specific predictors of incident HF in CCC. Therefore, we investigated in patients with CCC if age, sex, comorbidities, ECG, and echocardiographic characteristics, including 2D speckle tracking echocardiographic (STE) deformation analysis (*ε*) of left atrial (LA) and LV function, were associated with incident HF.

## 2 Patients and Methods

### 2.1 Design

This is a single‐center retrospective analysis of the occurrence of incident HF among patients with CCC from a longitudinal observational cohort study, which included patients recruited consecutively among those referred for echocardiograms between March 2010 and February 2014 and followed until June 2023 [11]. Inclusion criteria were patients aged between 18 and 80 years with chronic CD, both sexes, diagnosed by positivity in two distinct serological tests (enzyme‐linked immunosorbent assay and indirect immunofluorescence) [4], with CCC, according to the presence of changes in ECG as described by the Brazilian CD consensus criteria [4], but without HF. The ECG changes considered compatible with CCC were as follows [4]: complete right bundle branch block (RBBB), associated or not with a left anterior fascicular block (LAHB); frequent polymorphous or repetitive ventricular premature beats; nonsustained ventricular tachycardia; second‐ and third‐degree atrioventricular block; sinus bradycardia with heart rate <40 beats/min; sinus node dysfunction; complete left bundle‐branch block; atrial fibrillation; electric inactive area; and primary T wave changes. ECGs were analyzed by experienced cardiologists and classified using the Minnesota Code criteria, modified for CD [16].

### 2.2 Study Endpoint and Follow‐Up

The study endpoint was incident HF. The diagnosis of incident HF depended on the presence of symptoms and/or signs of HF and objective evidence of cardiac dysfunction obtained by echocardiography [17]. All patients were evaluated by cardiologists highly experienced in following patients with CCC, and HF diagnosis was confirmed by a second cardiologist who reviewed the medical records. Patients underwent medical visits at least four times a year and annual ECG and echocardiograms. Additional returns to the medical office and additional ECGs, 24‐hour Holter monitoring exams, and echocardiograms were determined by clinical status, complications, and treatment.

Patients were censored due to loss of follow‐up on the day of the last medical visit or due to death. All‐cause mortality was included as a competing event in statistical analyses because sudden death is the most frequent cause of death among patients with CCC and no HF [18]. Death was classified as related to CD (sudden death or stroke), due to other causes, such as cancer, trauma or co‐infections, or unknown cause. In the case of patients who did not return for medical appointments, mortality data were also retrieved from registries of death certificates available at the Department of Justice of the Rio de Janeiro state (http://www4.tjrj.jus.br/Portal‐Extrajudicial/CNO/). Living relatives were also contacted in order to obtain copies of death certificates in case the patient did not die during admission to our institutional hospital.

### 2.3 Echocardiography

Echocardiograms were performed and analyzed as previously described [11, 19]. Briefly, a phased‐array ultrasound system (Vivid 7, GE Medical Systems, Milwaukee, WI) equipped with M4S phased‐array and 1.5‐ to 4‐MHz four‐matrix‐array transducers was used to acquire the images and Echopac PC workstation software version 108.1.12 (GE Medical Systems) was used for imaging analyses. Cardiac dimensions, measured using M‐mode and 2D echocardiography, and Doppler measurements were obtained as recommended [20]. LV diastolic function was retrospectively classified as recommended [21]. LA *ε* was determined as previously described [19] using apical 4‐and 2‐chamber views. The onset of the P‐wave was used as the reference point, and LA conduit *ε* (LAScd), LA booster contraction *ε* (LASct), and LA reservoir *ε* (LASr) were obtained. In the case of patients with atrial fibrillation, the QRS was used as the reference point, and only LASr was obtained. LV longitudinal (LV‐GLS), LV circumferential (LV‐GCS), radial *ε* (LV‐GRS), LV torsion, and twist were analyzed as previously described [11, 19]. LV‐GLS and LV‐GCS are presented as absolute values.

### 2.4 Statistical Analysis

Continuous variables were expressed as mean ± standard deviation and categorical variables as absolute and percentage values. Kolmogorov–Smirnov tests provided support that continuous variables were normal, as *p* values were >0.10. Associations between studied variables and event‐free survival time were tested using univariate and multivariate competing‐risk survival regression with the Fine and Gray method to account for the possibility of competing risks for all‐cause mortality. A multivariate model 0 including sex, age, and clinical variables with univariate association with the study endpoint, as well as variables representing LV systolic and diastolic function and LA dimension, selected based on the largest Harrell's C‐index from univariate analyses was performed. Additional competing‐risk survival regression models were fitted for each 2D *ε* variable added to model 0 to evaluate the independent predictive value of the 2D *ε* variables. The Harrell's C‐indices for these models were compared to those of model 0 to determine whether the predictive value of each of these new models exceeded that of model 0. Missing data were handled by listwise deletion. Multicollinearity was assessed by calculation of the variance inflation factor (VIF) of the studied models.

Receiver operating characteristic (ROC) curves were generated to define optimal cutoff values for the variables with independent association with the study endpoint. The optimal cutoff obtained for a ROC curve corresponded to the maximum of the Youden index, defined as J = max[SE_i_ + SP_i_ − 1], where SE_i_ and SP_i_ are the sensitivity and specificity over all possible threshold values. Assessment of differences between areas under the ROC curve (AUC) was accomplished by pairwise comparison, as described by DeLong et al. [22]. Cumulative survival curves dichotomized at optimal ROC were constructed using the Kaplan–Meier method and compared using the log‐rank test.

Calculations were done using MedCalc version 20.113 (MedCalc Software, Mariakerke, Belgium) and Stata version 18.0 (StataCorp, College Station, TX). The null hypothesis was rejected at *p* < 0.05.

## 3 Results

### 3.1 Baseline Characteristics

A total of 176 patients with CCC and no HF diagnosis were included at baseline. From those, 141 patients (80.1%) presented normal LV ejection fraction, 31 patients (17.6%) presented mild LV systolic dysfunction, three patients (1.7%) presented moderate LV systolic dysfunction, and one patient (0.6%) presented severe LV systolic dysfunction at baseline.

The clinical, electrocardiographic, and echocardiographic patients’ characteristics at baseline are described in Table 1. Most patients were female, and hypertension and dyslipidemia were the most frequent comorbidities. The most common ECG abnormalities were RBBB, LAHB, and primary T wave changes. Most patients had normal LV diastolic and systolic function at baseline. Regarding right ventricular (RV) systolic function, only one patient (0.6%) presented RV systolic dysfunction at baseline based on RV peak systolic tricuspid annulus velocity (RV S’) and tricuspid annular plane excursion (TAPSE) analyses.

**TABLE 1:** Clinical and echocardiographic characteristics at baseline.

Variable	
**Age (years)**	53.9 ± 10.0
**Sex (men)**	72 (40.9)
**Body mass index (kg/m^2^)**	26.2 ± 4.0
**Hypertension**	63 (35.8)
**Diabetes mellitus**	7 (4.0)
**Dyslipidemia**	81 (46.0)
**Smoking**	12 (6.8)
**Electrocardiogram**	
RBBB	109 (61.9)
LBBB	4 (2.3)
LAHB	78 (44.3)
Primary T wave changes	57 (32.4)
Electric inactive areas	3 (1.7)
Low voltage	10 (5.7)
Atrial fibrillation	2 (1.1)
Cardiac device	21 (11.9)
Dual‐chamber pacemaker	19 (10.8)
Single‐chamber pacemaker	2 (1.1)
ICD	0
**2D echocardiogram**	
LA (cm)	3.8 ± 0.5
LA volume (mL/m^2^)	28.6 ± 9.7
LVd (cm)	5.4 ± 0.6
LVs (cm)	3.6 ± 0.8
LV ejection fraction (%)	61.6 ± 10.2
LV S’ (cm/s)	7.4 ± 1.8
RV S’ (cm/s)	13.0 ± 2.3
TAPSE (mm)	24.1 ± 4.4
PASP (mmHg)	30.6 ± 7.3
E/A ratio	1.1 ± 0.6
E’ (cm/s)	8.4 ± 2.8
A’ (cm/s)	9.6 ± 2.3
E/E’ ratio	9.1 ± 3.4
LV aneurysm	36 (20.4)
LV diastolic function	
Normal	120 (68.2)
Grade I	31 (17.6)
Grade II	19 (10.8)
Grade III	4 (2.2)
**Strain**	
LASct (%)	−12.6 ± 3.2
LAScd (%)	12.7 ± 5.1
LASr (%)	25.2 ± 5.9
LV‐GLS (%)	16.8 ± 3.5
LV‐GCS (%^a^)	16.9 ± 4.9
LV‐GRS (%^a^)	37.3 ± 13.9
Twist (^0^)	10.2 ± 6.1
Torsion (^0^/cm)	1.25 ± 0.76

*Note*: Values are mean ± SD or *n*(%).

Abbreviations: A, peak late wave diastolic filling velocity; A’, peak late diastolic mitral annulus velocity; E, peak early wave diastolic filling velocity; E’, peak early diastolic mitral annulus velocity; *ε*, strain; GCS, global circumferential *ε*; GLS, global longitudinal *ε*; GRS, global radial *ε*; ICD, implantable cardioverter defibrillator; LAHB, left anterior hemiblock; LASct, LA booster contraction *ε*; LAScd, LA conduit *ε*; LASr, LA reservoir *ε*; LBBB, left bundle branch block; LVd, LV end‐diastolic diameter; LVs, LV end‐systolic diameter; LV S’, peak systolic mitral annulus velocity; PASP, pulmonary artery systolic pressure; RBBB, right bundle branch block; RV S’, peak systolic tricuspid annulus velocity; TAPSE, tricuspid annular plane excursion.

^a^ LV‐GLS and LV‐GCS are presented as absolute values.

Poor imaging quality was the reason to exclude six patients (3.4%) from LV‐GLS analysis, nine patients (5.1%) from LV‐GCS and LV‐GRS analyses, and 15 patients (8.5%) from LV torsion analysis. LA *ε* analyses were feasible in all but two (1.1%) participants. The intra‐ and interobserver variabilities for LA *ε* [19] and LV *ε* [23] had already been published.

### 3.2 Survival Free of Incident HF

A total of 42 (23.9%) patients presented incident HF during a mean follow‐up of 8.8 ± 3.6 years, representing an event rate of 27.0 cases/1000 patient‐years (CI 95% 19.99–36.60 cases/1000 patients‐years). From those without global or segmental LV wall motion changes at baseline, nine out of 91 (9.9%) presented incident HF, while from those with global or segmental LV wall motion changes at baseline, 33 out of 85 (38.8%) presented incident HF (HR = 5.2, 95% CI: 2.8 to 9.6, *p* < 0.0001). Patients lost to follow‐up (*n* = 21) were censored from analysis on the date of their last medical appointment. The mean LV ejection fraction at baseline was similar between patients who were lost or who were not lost to follow‐up: (62.9% ± 9.8% vs. 61.5% ± 10.3%, *p* = 0.55). The mean LV ejection fraction determined after HF diagnosis decreased to 36.0% ± 10.3%. The classification of the cases of incident HF based on LV ejection fraction were as follows, 31 (73.8%) patients presented HFrEF, nine (21.4%) patients presented HFmrEF, and two (4.8%) patients presented HFpEF.

A total of 24 (13.6%) patients were censored due to all‐cause mortality. The cause of death was related to CD in 12 patients (sudden death in 10 patients and stroke in two patients), due to other causes in four patients (COVID‐19 [*n* = 1], uterine cancer [*n* = 1], liver cirrhosis due to chronic hepatitis C virus infection [*n* = 1], and in‐hospital complications after colectomy [*n* = 1]), and unknown in eight patients. We included those deaths as competing events in survival analyses as those patients could have developed HF in case they had not died.

In the univariate models, diabetes mellitus, larger LA dimension, larger LV diameters, lower LV ejection fraction, lower LV peak systolic mitral annulus velocity, lower peak early (E’) and late diastolic mitral annulus velocities, and higher peak early wave diastolic filling to E’ velocities ratio were associated with incident HF (Table 2). All LA and LV *ε* parameters were associated with incident HF in the univariate analyses.

**TABLE 2:** Univariate associations between clinical, electrocardiographic, and echocardiographic characteristics with incident HF.

Variable	HR	95% CI	*p* values	Harrell's C‐index (95% CI)
**Age (years)**	1.02	0.98–1.05	0.38	0.55 (0.42–0.68)
**Sex (men)**	1.42	0.78–2.60	0.25	0.54 (0.44–0.64)
**Body mass index (kg/m^2^)**	1.02	0.94–1.11	0.64	0.51 (0.40–0.62)
**Hypertension**	1.33	0.72–2.45	0.36	0.56 (0.46–0.67)
**Diabetes mellitus**	4.47	1.43–13.9	0.01	0.55 (0.48–0.62)
**Dyslipidemia**	1.05	0.57–1.91	0.88	0.51 (0.40–0.61)
**Smoking**	0.29	0.04–2.16	0.23	0.51 (0.47–0.56)
**Electrocardiogram**				
RBBB	1.33	0.69–2.55	0.40	0.50 (0.40–0.60)
LBBB	4.01	0.74–21.5	0.10	0.50 (0.49–0.50)
LAHB	1.53	0.83–2.84	0.17	0.59 (0.49–0.69)
Primary T wave changes	0.85	0.44–1.64	0.63	0.52 (0.43–0.61)
Electric inactive areas	1.79	0.17–18.7	0.49	0.52 (0.47–0.57)
Low voltage	0.79	0.17–3.61	0.76	0.50 (0.49–0.51)
Cardiac device	1.59	0.71–3.56	0.26	0.55 (0.46–0.64)
**2D echocardiogram**				
LA (cm)	3.00	1.76–5.11	<0.001	0.72 (0.63–0.81)
LA volume (mL/m^2^)	1.03	1.002–1.06	0.03	0.70 (0.60–0.80)
LVd (cm)	2.82	2.04–3.88	<0.001	0.65 (0.53–0.77)
LVs (cm)	2.25	1.77–2.86	<0.001	0.71 (0.60–0.81)
LV ejection fraction (%)	0.94	0.92–0.97	<0.001	0.76 (0.68–0.84)
LV S’ (cm/s)	0.55	0.44–0.69	<0.001	0.70 (0.60–0.80)
RV S’ (cm/s)	0.88	0.76–1.03	0.11	0.56 (0.44–0.69)
TAPSE (mm)	0.96	0.89–1.03	0.27	0.51 (0.40–0.61)
PASP (mmHg)	1.03	0.99–1.07	0.17	0.66 (0.55–0.77)
E/A ratio	1.27	0.63–2.56	0.51	0.50 (0.36–0.65)
E’ (cm/s)	0.76	0.66–0.85	<0.001	0.67 (0.55–0.79)
A’ (cm/s)	0.77	0.66–0.91	0.001	0.70 (0.60–0.80)
E/E’ ratio	1.13	1.05–1.21	0.001	0.71 (0.61–0.81)
LV aneurysm	1.75	0.89–3.46	0.10	0.52 (0.43–0.61)
**Strain**				
LASct (%)	1.11	1.00–1.24	0.049	0.72 (0.63–0.82)
LAScd (%)	0.91	0.86–0.97	0.002	0.59 (0.47–0.71)
LASr (%)	0.89	0.85–0.94	<0.001	0.71 (0.61–0.82)
LV‐GLS (%)	0.79	0.72–0.86	<0.001	0.76 (0.70‐ 0.82)
LV‐GCS (%)	0.83	0.78–0.89	<0.001	0.75 (0.69–0.82)
LV‐GRS (%)	0.95	0.93–0.98	<0.001	0.66 (0.53–0.78)
Twist (^0^)	0.92	0.89–0.96	<0.001	0.70 (0.60–0.80)
Torsion (^0^/cm)	0.52	0.37–0.73	<0.001	0.68 (0.58–0.78)

Abbreviations: A, peak late wave diastolic filling velocity; A’, peak late diastolic mitral annulus velocity; E, peak early wave diastolic filling velocity; E’, peak early diastolic mitral annulus velocity; *ε*, strain; GCS, global circumferential *ε*; GLS, global longitudinal *ε*; GRS, global radial *ε*; HF, heart failure; HR, hazard ratio; LA, left atrial; LAHB, left anterior hemiblock; LASct, LA booster contraction *ε*; LAScd, LA conduit *ε*; LASr, LA reservoir *ε*; LBBB, left bundle branch block; LV, left ventricular; LVd, LV end‐diastolic diameter; LVs, LV end‐systolic diameter; LV S’, peak systolic mitral annulus velocity; PASP, pulmonary artery systolic pressure; RBBB, right bundle branch block; RV, right ventricular; RV S’, peak systolic tricuspid annulus velocity; TAPSE, tricuspid annular plane excursion.

**FIGURE 1:**
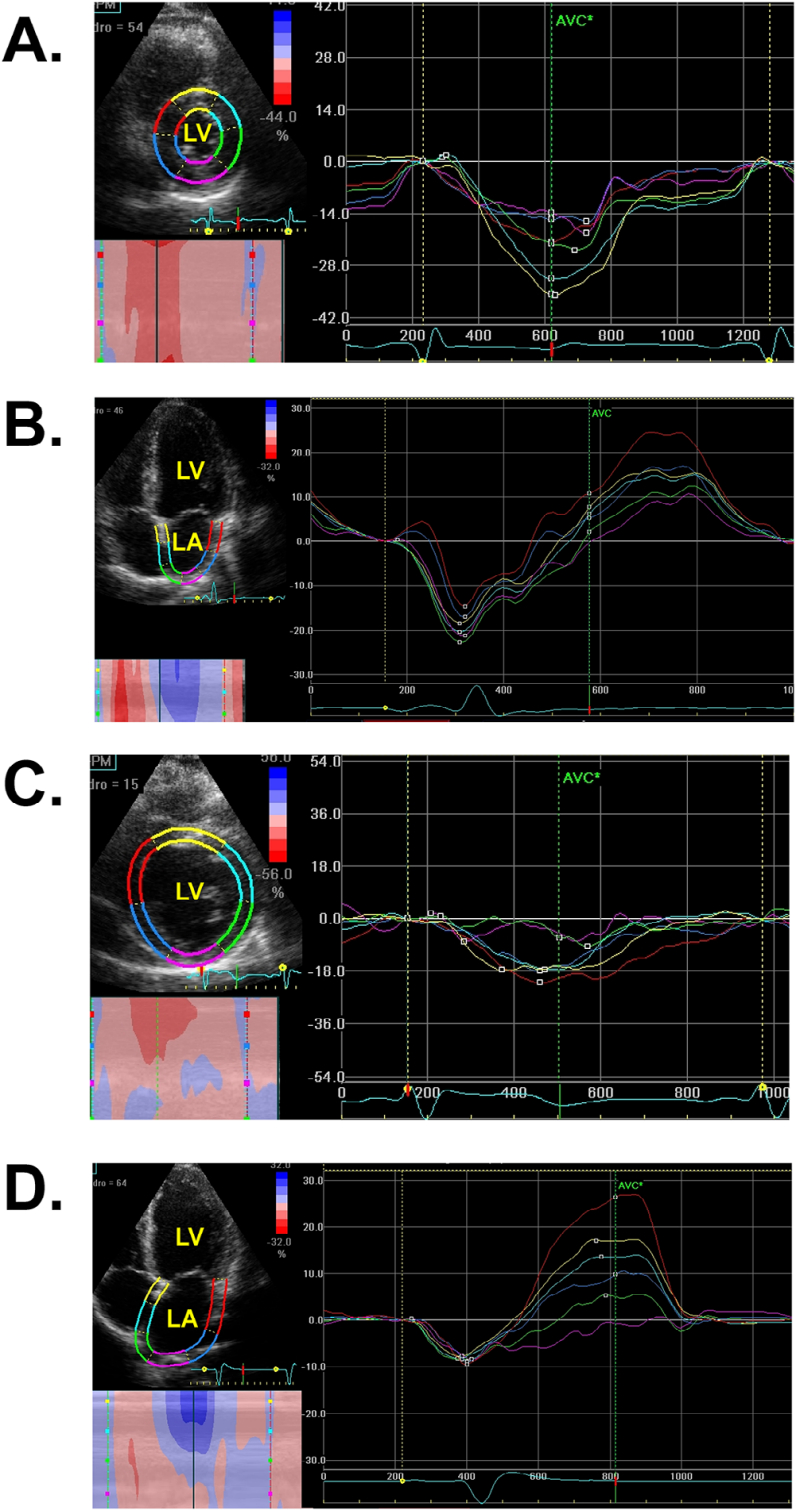
Echopac PC workstation software analysis of LV‐CS and LA *ε*. Note the higher absolute LV‐CS (A) and LASct (B) values from patients who did not present incident HF compared to those who presented HF (C and D). HF, heart failure; LA, left atrial; LASct, LA booster contraction *ε*; LV, left ventricular; LV‐CS, LV circumferential *ε*.

A multivariate model 0 with diabetes mellitus, age, sex, LA diameter, LV ejection fraction and E’ velocity revealed that diabetes mellitus (HR 4.91, 95% CI 1.67–14.4, *p* = 0.004), LV ejection fraction (HR 0.96, 95% CI 0.93–0.99, *p* = 0.022), and E’ velocity (HR 0.79, 95% CI 0.67–0.95, *p* = 0.01) were independently associated with incident HF. For each 2D LA and LV *ε* variables, a multivariate model was created including the variable of interest adjusted for the variables of the model 0 and all‐cause mortality in order to verify their independent association with the study endpoint. LASct and LV‐GCS were independent predictors of incident HF (Table 3; Figure 1). The full multivariate analyses are presented in Tables S1–S3. The VIF values of the multivariable models ranged from 1.50 to 1.77 and the Harrell's C‐indexes of the models with the variables of interest did not differ significantly from the model 0 Harrell's C‐index (0.82 [95% CI 0.77–0.87]).

**TABLE 3:** Multivariate models for the association between 2D strain parameters and incident HF.

	Adjusted analyses			
Variable	HR	95% CI	*p* values	Harrell's C‐index (95% CI)
LASct (%)	1.14	1.02–1.28	0.022	0.82 (0.77–0.88)
LAScd (%)	1.01	0.94–1.09	0.71	0.82 (0.77–0.87)
LASr (%)	0.94	0.87–1.01	0.09	0.81 (0.86–0.86)
LV‐GLS (%)	0.88	0.77–1.02	0.09	0.82 (0.77–0.88)
LV‐GCS (%)	0.85	0.78–0.93	<0.001	0.83 (0.78–0.88)
LV‐GRS (%)	0.97	0.94–1.00	0.085	0.82 (0.76–0.87)
Twist (^0^)	0.96	0.90–1.01	0.12	0.82 (0.77–0.87)
Torsion (^0^/cm)	0.72	0.44–1.17	0.18	0.82 (0.77–0.87)

*Notes*: Multivariate models were adjusted for age, sex, LV ejection fraction, E’ velocity, LA diameter, presence of diabetes mellitus, and all‐cause mortality.

Abbreviations: E’, peak early diastolic mitral annulus velocity; *ε*, strain; GCS, global circumferential *ε*; GLS, global longitudinal *ε*; GRS, global radial *ε*; HF, heart failure; HR, hazard ratio; LA, left atrial; LASct, LA booster contraction *ε*; LAScd, LA conduit *ε*; LASr, LA reservoir *ε*; LV, left ventricular.

**FIGURE 2:**
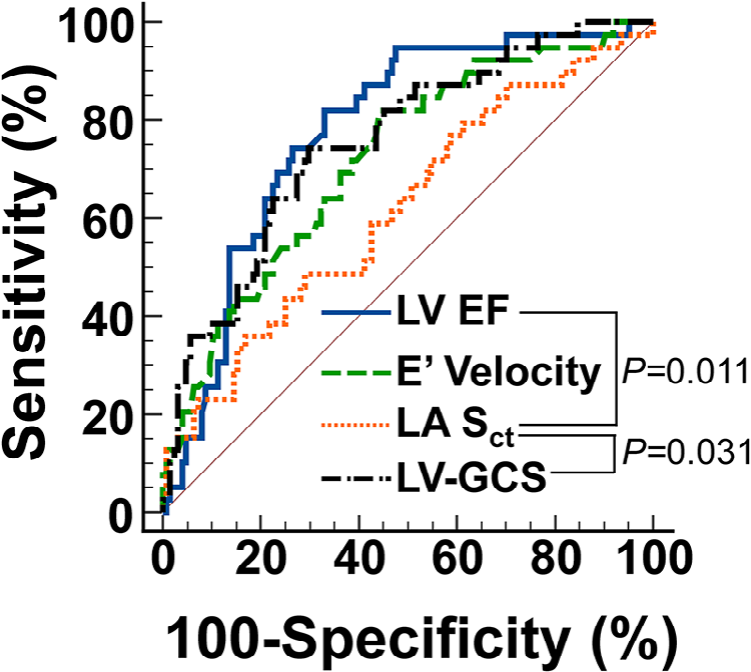
ROC curves generated for independent predictors of incident heart failure. The AUC for LASct (0.60) was lower than the AUC for LV ejection fraction (0.76, *p* = 0.011) and LV‐GCS (0.76, *p* = 0.03), while it did not differ significantly from AUC for E’ velocity (AUC 0.71, *p* = 0.18). AUC, area under ROC curve; *ε*, strain; E’, peak early diastolic mitral annulus velocity; LASct, LA booster contraction *ε*; LV, left ventricular; LV‐GCS, LV circumferential *ε*; ROC, receiver operating characteristic.

Optimal cutoff values to predict incident HF for LV ejection fraction was 60.6% (AUC 0.76, sensitivity 80.9%, specificity 66.4%, *p* < 0.0001), for E’ velocity was 8.5 cm/s (AUC 0.71, sensitivity 80.9%, specificity 53.7%, *p* < 0.0001), for LASct was −11.40% (AUC 0.60, sensitivity 46.3%, specificity 71.0%, *p* = 0.05), and for LV‐GCS was 15.5% (AUC 0.76, sensitivity 75.0%, specificity 70.1%, *p* < 0.0001). The AUC for LASct was lower than the AUC for LV ejection fraction (*p* = 0.011) and LV‐GCS (*p* = 0.03; Figure 2).

The variables with independent association with incident HF in model 0 (diabetes mellitus, LV ejection fraction, E’ velocity) plus LV‐GCS and LASct were entered in a multivariate logistic regression model in order to verify if their combination could improve the predictive value. The model revealed that E’ velocity (OR = 0.77, 95% CI 0.64–0.94, *p* = 0.009), LV‐GCS (OR = 0.87, 95% CI 0.77–0.99, *p* = 0.031), and LASct (OR = 1.17, 95% CI 1.02–1.34, *p* = 0.027) remained independently associated with the studied event. The AUC of the multivariate model was 0.83 (95% CI 0.76–0.88; sensitivity 79.5%; specificity 75.8%; *p* < 0.0001) and was higher than the one for E’ velocity (*p* = 0.012), LASct (*p* < 0.0001), and LV‐GCS (*p* = 0.03), but not significantly higher than the one for LV ejection fraction (*p* = 0.17).

**FIGURE 3:**
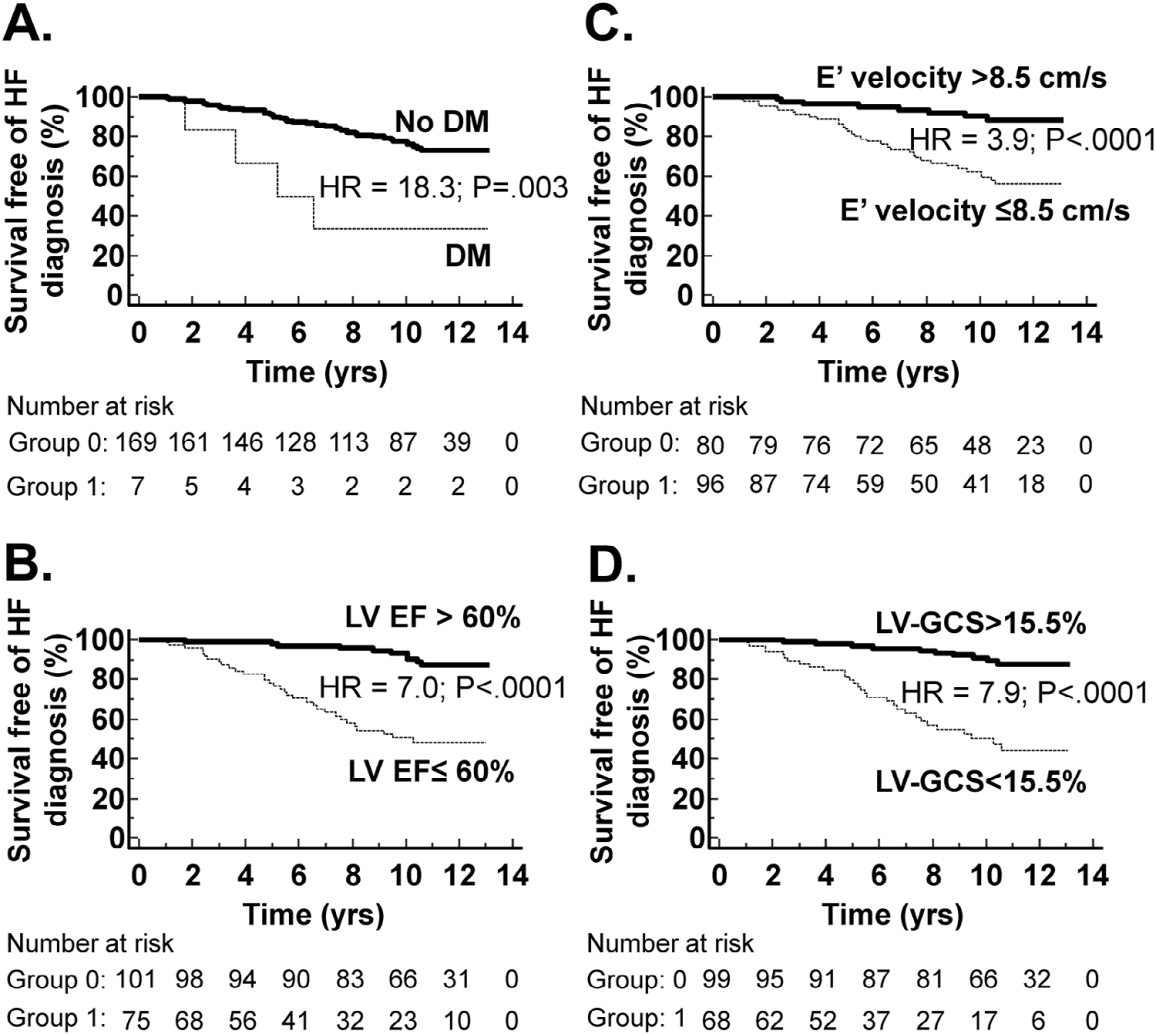
Survival curve free of incident HF. Kaplan–Meier survival curves are free of incident HF according to the presence of diabetes mellitus (A), LV EF ≤60% (B), E’ velocity ≤8.5 cm/s (C), and absolute LV‐GCS <15.5% (D). DM, diabetes mellitus; *ε*, strain; E’, peak early diastolic mitral annulus velocity; EF, ejection fraction; HF, heart failure; HR, hazard ratio; LV, left ventricular; LV‐GCS, LV circumferential *ε*.

Survival curves free of incident HF were constructed dichotomized according to the optimal cutoff. Presence of diabetes mellitus at baseline (HR = 18.35, 95% CI:2.62 to 128.3, *p* = 0.003; Figure 3A), LV ejection fraction ≤60% (HR = 7.00, 95% CI: 3.67 to 13.36, *p* < 0.0001; Figure 3B), E’ velocity ≤8.5 cm/s (HR = 3.86, 95% CI:2.10 to 7.09, *p* < 0.0001; Figure 3C), and LV‐GCS absolute value <15.5% (HR = 7.89, 95% CI:4.02 to 15.49, *p* < 0.0001; Figure 3D) were associated with less survival free of incident HF.

## 4 Discussion

To the best of our knowledge, this is the first study to describe the incidence of HF and its predictors in patients with CCC. We found that multivariate models including LV systolic parameters and STE‐derived parameters had good discriminatory power to identify patients at risk of incident HF. Importantly, the incidence of HF in patients with CCC described by us is higher than the cumulative incidence of HF described in a meta‐analysis (0.97%) [13], in stable survivals of myocardial infarction (6.3%) [24], or in elderly (8.0%) [25]. This fact is another hallmark of the worse prognosis that patients with CD face compared to patients with other cardiopathies. Other important aspect is that the majority of patients with CCC progress to HF with reduced ejection fraction.

Although several clinical risk factors for incident HF, such as age [13, 24, 26, 27], male sex [13, 27] hypertension [13, 24, 26, 27], diabetes [13, 28], dyslipidemia [26], smoking [13, 26, 27], and body mass index [13, 26, 27], are described in the general population or in other HF etiologies, only diabetes was associated with incident HF in our study with patients with CCC. Therefore, our findings indicate the need for a specific predictive model for incident HF in CCC to be used in clinical practice.

2D echocardiographic parameters were previously described as incident HF predictors, including LV ejection fraction [13, 24]. Moreover, reduced LV ejection fraction, abnormal E/A ratio, enlarged LA, and increased LV mass, were described to improve the 5‐year HF risk prediction provided by the clinical Health Aging and Body Composition HF risk score in the elderly [25]. More recently, individuals at risk for HF, LV‐GLS, and diastolic function were able to reclassify 61% of the of the intermediate‐risk group according to the 4‐year Atherosclerosis Risk in Communities HF risk score into the low‐risk group [29]. Accordingly, in our article parameters of LV systolic and diastolic function were independent predictors of incident HF in patients with CCC.

Regarding STE‐derived parameters, LASct and LV‐GCS were independent predictors of incident HF after adjusting for all‐cause mortality, age, sex, diabetes mellitus, LV ejection fraction, E’ velocity, and LA dimension. LV‐GLS was described as a predictor of incident HF by others [29], but in our paper, LV‐GLS was associated with incident HF in univariate analysis and had a borderline association in multivariate analysis. The possible reason for LV‐GCS to be associated with incident HF independent from LV ejection fraction while the same was not observed with LV‐GLS may be related to the fibrosis pattern in early stages of CCC, which is predominantly midwall and not subendocardial [23, 30]. LV‐GLS is more associated with endocardial myocardial function, while LV‐GCS is more related with epicardial myocardial function [31].

Regarding LA function, few studies addressed LA function as a predictor of incident HF [32, 33, 34]. Specifically, all components of LA *ε* were independently associated with the composite of incident HF hospitalization or all‐cause death in older adults [33]. In the present article, LASct, LA booster function, was associated with incident HF after multivariate analyses. A decrease in LA contractile function can prompt an increase in LA pressure and a decrease in cardiac output, which can ultimately lead to HF symptoms. Importantly, we have previously described LASct as a predictor of cardiovascular events in patients with CD [35], and LASct was an independent predictor of cardiovascular death and HF hospitalization among patients with HFrEF [36].

### 4.1 Clinical Implications

Our study is the first to describe the incidence and parameters associated with incident HF in chronic CD. As HF is one of the main complications and cause of death in CCC [4, 5], this knowledge can be very useful to plan the follow‐up of these patients and to identify those who might benefit of early pharmacological and non‐pharmacological interventions. The echocardiographic variables associated with incident HF in model 0 are routinely obtained in everyday clinical practice and can be easily incorporated in future algorithms designed for the clinical follow‐up of patients with CCC. The LA and LV *ε* parameters are not readily obtained in most echocardiographic machines available in endemic areas, and further studies are warranted to validate the proposed cutoff values in independent populations and to evaluate the cost‐benefit of the implementation of this technology.

### 4.2 Limitations

Our study limitations include a low number of clinical events, absence of external validation of our findings, use of the same software to measure LV and LA *ε* instead of a dedicated software for LA *ε*, and use of the onset of the P‐wave instead of the QRS as the reference frame set to zero LA *ε*. However, at the time the echocardiograms were obtained, both methods to evaluate LA *ε* were equally adopted [37]. Another limitation is that B‐type natriuretic peptide measurements were not routinely available at our institution during the study period.

## 5 Conclusion

While most clinical parameters were not associated with incident HF in patients with CCC, echocardiographic parameters, such as LV ejection fraction, E’ velocity, LV‐GCS, and LASct, can be useful to identify those at risk of incident HF. This knowledge can be very useful for planning the care and follow‐up of patients with CCC.

## Ethics Statement

The study was approved by the Evandro Chagas National Institute of Infectious Diseases ethical committee under numbers 55/2009 on November 9, 2009 and 5.701.077 on October 14, 2022. This study conformed to standards applied by the Brazilian National Committee for Research Ethics and to the ethical guidelines of the Declaration of Helsinki and its amendments.

## Consent

All subjects gave written informed consent before their participation.

## Conflicts of Interest

The authors declare no conflicts of interest.

## Supporting information



Supporting Information

## Data Availability

The data underlying this article will be shared on reasonable request to the corresponding author.
