# Supplementary material for: Predictors of Incident Heart Failure in Patients With Chronic Chagas Disease Cardiomyopathy
Source: Echocardiography. 2025 Apr 28;42(5):e70163. doi: 10.1111/echo.70163 (PMC12036955; doi:10.1111/echo.70163)
Supplement: Supplementary file 1 — Supporting Information [file ECHO-42-e70163-s001.docx]

**Table S1**.Predictors of the study endpoint in multivariate analysis models adjusted for competing risks.

|  | **Adjusted Analyses** | | | | | | | | | | | |
| --- | --- | --- | --- | --- | --- | --- | --- | --- | --- | --- | --- | --- |
|  | **Model 0**  **n= 176** | | | **Model A –** LA Sct  **n= 172** | | | **Model B –** LA Scd  **n= 174** | | | **Model C –** LA Sr  **n=174** | | |
| **Variable** | **HR** | **95% CI** | **P value** | **HR** | **95% CI** | **P value** | **HR** | **95% CI** | **P values** | **HR** | **95% CI** | **P value** |
| Age, years | 0.97 | 0.92-1.02 | 0.23 | 0.94 | 0.89-0.99 | 0.016 | 0.95 | 0.90-1.01 | 0.086 | 0.94 | 0.89-0.99 | 0.042 |
| Sex, male | 0.88 | 0.43-1.80 | 0.72 | 0.85 | 0.40-1.80 | 0.67 | 0.71 | 0.33-1.55 | 0.39 | 0.81 | 0.37-1.77 | 0.60 |
| Diabetes mellitus | 4.91 | 1.67-14.4 | 0.004 | 6.86 | 2.52-18.71 | <0.001 | 4.94 | 1.63-14.95 | 0.005 | 5.29 | 1.87-14.98 | 0.002 |
| LA diameter, mm | 1.91 | 0.97-3.76 | 0.06 | 1.77 | 0.91-3.44 | 0.095 | 2.40 | 1.18-4.86 | 0.015 | 2.07 | 1.04-4.10 | 0.038 |
| LV EF, % | 0.96 | 0.93-0.99 | 0.022 | 0.97 | 0.94-1.00 | 0.064 | 0.96 | 0.93-0.99 | 0.014 | 0.97 | 0.94-0.99 | 0.046 |
| E’ velocity | 0.80 | 0.67-0.95 | 0.011 | 0.69 | 0.58-0.82 | <0.001 | 0.75 | 0.61-0.91 | 0.014 | 0.78 | 0.66-0.93 | 0.005 |
| **Variable of Interest** | - | - | - | 1.14 | 1.02-1.28 | 0.022 | 1.01 | 0.94-1.09 | 0.71 | 0.94 | 0.87-1.01 | 0.09 |
| **Harrell’s C-index (95% CI)** | 0.82 (0.77-0.87) | | | 0.82 (0.77-0.88) | | | 0.82 (0.77-0.87) | | | 0.81 (0.86-0.86) | | |

E’, peak early diastolic mitral annulus velocity; EF, ejection fraction; *ε*, strain; HR, hazard ratio; LA, left atrial; LASct, LA booster contraction *ε*; LAScd, LA conduit *ε*; LASr, LA reservoir *ε*; LV, left ventricular.

**Table S2**. Predictors of the study end-point in multivariable analysis models adjusted for competing risks.

|  | **Adjusted Analyses** | | | | | | | | | | | |
| --- | --- | --- | --- | --- | --- | --- | --- | --- | --- | --- | --- | --- |
|  | **Model D-** LV-GLS  **n= 170** | | | **Model E –** LV-GCS  **n=167** | | | **Model F –** LV-GRS  **n=167** | | | **Model G -** Peak Twist  **n= 161** | | |
| **Variable** | **HR** | **95% CI** | **P value** | **HR** | **95% CI** | **P value** | **HR** | **95% CI** | **P value** | **HR** | **95% CI** | **P value** |
| Age, years | 0.96 | 0.90-1.01 | 0.13 | 0.95 | 0.90-1.00 | 0.052 | 0.96 | 0.91-1.01 | 0.10 | 0.96 | 0.91-1.01 | 0.105 |
| Sex, male | 0.79 | 0.36-1.75 | 0.56 | 0.64 | 0.32-1.30 | 0.22 | 0.90 | 0.43-1.87 | 0.77 | 0.98 | 0.46-2.10 | 0.96 |
| Diabetes mellitus | 5.59 | 1.85-16.89 | 0.002 | 6.88 | 2.40-19.72 | <0.001 | 7.23 | 2.51-20.82 | <0.001 | 5.01 | 1.72-14.59 | 0.003 |
| LA diameter, mm | 2.36 | 1.17-4.78 | 0.017 | 2.61 | 1.32-5.18 | 0.006 | 2.40 | 1.27-4.53 | 0.007 | 2.15 | 1.12-4.13 | 0.021 |
| LV EF, % | 0.98 | 0.94-1.03 | 0.50 | 1.00 | 0.96-1.04 | 0.92 | 0.98 | 0.94-1.02 | 0.34 | 0.97 | 0.94-1.01 | 0.13 |
| E’ velocity | 0.81 | 0.67-0.97 | 0.022 | 0.79 | 0.68-0.94 | 0.006 | 0.81 | 0.68-0.96 | 0.015 | 0.80 | 0.67-0.96 | 0.017 |
| **Variable of Interest** | 0.88 | 0.77-1.02 | 0.09 | 0.85 | 0.78-0.93 | <0.001 | 0.97 | 0.94-1.00 | 0.085 | 0.96 | 0.90-1.01 | 0.12 |
| **Harrell’s C-index (95% CI)** | 0.82 (0.77-0.88) | | | 0.83 (0.78-0.88) | | | 0.82 (0.76-0.87) | | | 0.82 (0.77-0.87) | | |

E’, peak early diastolic mitral annulus velocity; EF, ejection fraction; *ε*, strain; GCS, global circumferential *ε*; GLS, global longitudinal *ε*; GRS, global radial *ε*; HR, hazard ratio; LA, left atrial; LV, left ventricular.

**Table S3**. Predictors of the study end-point in multivariable analysis models adjusted for competing risks.

|  | **Adjusted Analysis** | | |
| --- | --- | --- | --- |
|  | **Model H-** Peak Torsion  **n= 161** | | |
| **Variable** | **HR** | **95% CI** | **P value** |
| Age, years | 0.96 | 0.92-1.01 | 0.12 |
| Sex, male | 0.94 | 0.44-1.98 | 0.87 |
| Diabetes mellitus | 4.93 | 1.69-14.40 | 0.003 |
| LA diameter, mm | 2.15 | 1.12-4.14 | 0.021 |
| LV EF, % | 0.97 | 0.94-1.01 | 0.13 |
| E’ velocity | 0.81 | 0.67-0.96 | 0.019 |
| **Variable of Interest** | 0.72 | 0.44-1.17 | 0.18 |
| **Harrell’s C-index (95% CI)** | 0.82 (0.77-0.87) | | |

E’, peak early diastolic mitral annulus velocity; EF, ejection fraction; LA, left atrial; LV, left ventricular.
